# Defects in bilayer silica and graphene: common trends in diverse hexagonal two-dimensional systems

**DOI:** 10.1038/srep03482

**Published:** 2013-12-16

**Authors:** Torbjörn Björkman, Simon Kurasch, Ossi Lehtinen, Jani Kotakoski, Oleg V. Yazyev, Anchal Srivastava, Viera Skakalova, Jurgen H. Smet, Ute Kaiser, Arkady V. Krasheninnikov

**Affiliations:** 1 COMP/Department of Applied Physics, Aalto University School of Science, P.O. Box 11100, 00076 Aalto, Finland; 2 Electron Microscopy Group of Materials Science, University of Ulm, Germany 89081; 3 Department of Physics, University of Helsinki, P.O. Box 43, 00014 Helsinki, Finland; 4 Faculty of Physics, University of Vienna, Boltzmanngasse 5, 1190 Wien, Austria; 5 Institute of Theoretical Physics, École Polytechnique Fédérale de Lausanne (EPFL), CH-1015 Lausanne, Switzerland; 6 Max Planck Institute for Solid State Research, Stuttgart, Germany 70569; 7 Department of Physics, Banaras Hindu University, Varanasi, India 221005

## Abstract

By combining first-principles and classical force field calculations with aberration-corrected high-resolution transmission electron microscopy experiments, we study the morphology and energetics of point and extended defects in hexagonal bilayer silica and make comparison to graphene, another two-dimensional (2D) system with hexagonal symmetry. We show that the motifs of isolated point defects in these 2D structures with otherwise very different properties are similar, and include Stone-Wales-type defects formed by structural unit rotations, flower defects and reconstructed double vacancies. The morphology and energetics of extended defects, such as grain boundaries have much in common as well. As both sp^2^-hybridised carbon and bilayer silica can also form amorphous structures, our results indicate that the morphology of imperfect 2D honeycomb lattices is largely governed by the underlying symmetry of the lattice.

The family of two-dimensional (2D) crystals was recently joined by one of the most abundant substances on earth, silica. Two different polymorphs of this material, both with hexagonal symmetry, have been synthesised on various metal substrates^1^,^2^,^3^,^4^,^5^. While one of the forms is a single layer of the tetrahedra-shaped structural units covalently bonded to the metal^1^,^2^,^4^, the other is a fully saturated bilayer structure, which is only weakly bound to the substrate by van der Waals interaction^2^,^3^. Apart from being the thinnest gate dielectric oxide layer and support in catalysis^6^, the 2D silica polymorphs have been demonstrated to be suitable for isolation of graphene from a metal substrate^7^ by intercalating Si and O atoms between the two systems. Moreover, the vitreous state of the system, where the structural units form a disordered network, has sparked considerable interest in the context of determining the atomic arrangement of a glassy structure^2^,^3^,^8^. This is of fundamental scientific interest since, although scanning tunneling microscopy and atomic force microscopy can be used to study their surfaces, direct imaging of conventional glasses in bulk at atomic resolution remains impossible.

Experiments have shown^6^ that the amount of disorder in 2D silica can continuously vary starting from isolated point defects and grain boundaries in crystalline systems up to completely amorphous structures, similar to graphene^9^. The relationship between the structures of the crystalline and amorphous systems can be understood in terms of bond rotations, similar to Stone-Wales (SW) transformations in sp^2^-hybridised carbon systems^10^. Observations of striking similarities in defect motives in graphene and 2D silica^3^,^5^,^8^, two systems of potential high technological importance with different properties, and the existence of a 2D carbon analog^9^ of amorphous silica gives rise to a question about the nature of defects in these hexagonal 2D systems: despite their very different bonding properties, do the defects in these two 2D systems behave in a similar way?

In this study, we investigate the atomic structure and properties of point defects and grain boundaries in 2D hexagonal bilayer silica (HBS) and graphene, using a combination of aberration corrected high resolution transmission electron microscopy (AC-HRTEM) and atomistic calculations based on the density functional theory (DFT) and classical force field (CFF) approaches. While defects in graphene have extensively been studied, see e.g. Ref. 11 for an overview, the nature of defects in HBS is so far less well known. Although graphene and HBS are the only 2D hexagonal materials that have been extensively studied so far, recent progress in this area, as indicated by the development of epitaxial atomically thin silicon structures^12^, epitaxial hexagonal transition metal networks^13^, a large number of 2D compounds with trigonal symmetry, such as h-BN and the transition metal dichalcogenides^14^,^15^ as well as computational discoveries of new 2D compounds^16^,^17^, suggests that other nearly free-standing hexagonal 2D materials can be created, which provides additional stimulus to study common trends in defect energetics and morphology in 2D systems.

## Results

**Figure 1:**
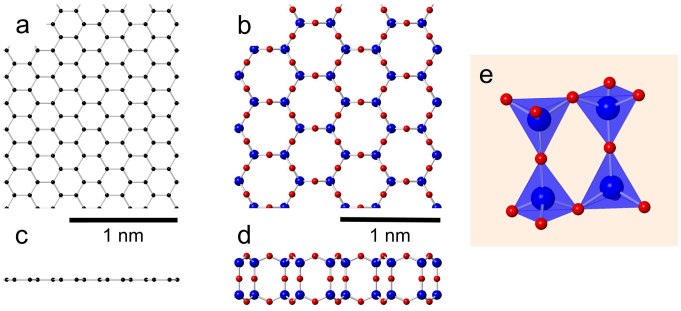
Atomic structure of the two 2D structures. (a) Graphene, top-view. (b) Texagonal bilayer silica (HBS), top-view. (c) Graphene, side-view. (d) HBS, side-view. (e) Tetrahedral structural units of the HBS structure.

While the atomic structure of graphene is simple and well known, see Fig. 1(a), the HBS system is more involved. It can be constructed by arranging four oxygen atoms in a tetrahedron surrounding a single Si atom and letting the oxygen cages share corners in a hexagonal network, as shown in Figure 1(e). This arrangement is stoichiometric and saturates all covalent bonds, thus leaving no further opportunities for chemical bonding to the surface, which accounts for the weak substrate interaction previously reported^2^,^3^. If oxygen atoms (the red balls in Fig. 1 are omitted, the atomic network (the top view) is similar to graphene, exhibiting the same hexagonal symmetry.

### Point defects

**Figure 2:**
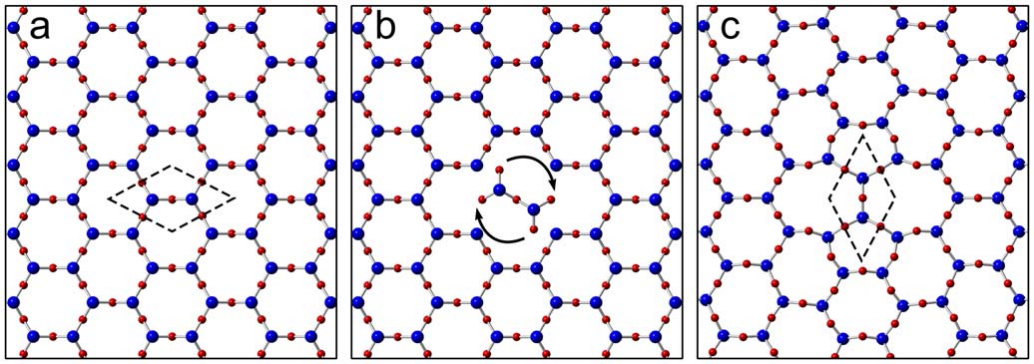
Stone-Wales transformation in hexagonal bilayer silica. (a) The pristine lattice is transformed (b) by means of rotation of a pair of structural units into (c) the final Stone-Wales defect. Note that this is a schematic illustration to visualise the transition between the initial and final topologies and that (b) does not necessarily represent an actual intermediate state.

**Figure 3:**
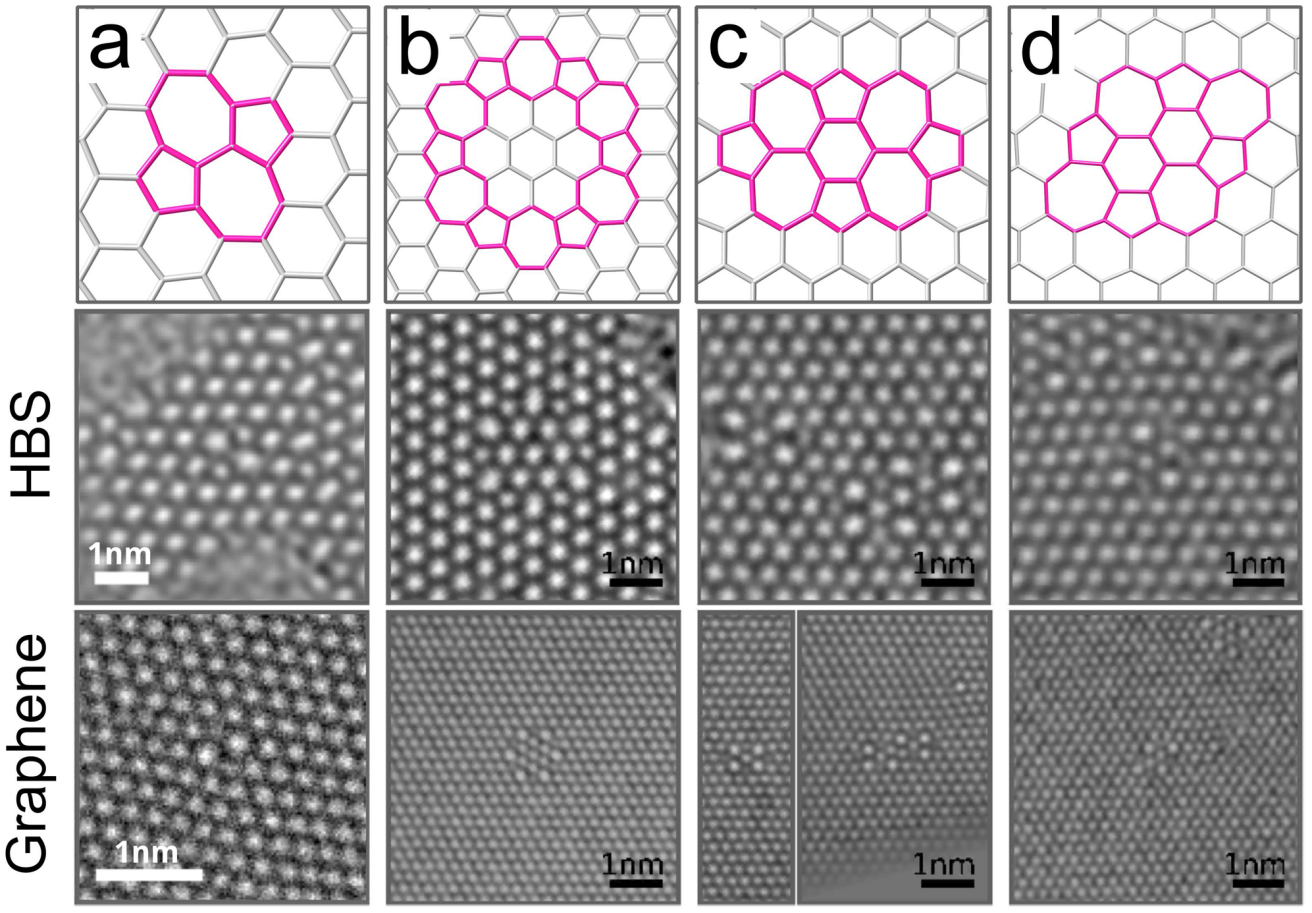
80 kV AC-HRTEM images of isolated defects. Atomic models (top row) and AC-HRTEM images of isolated defects in HBS and graphene (middle and bottom rows, respectively). Stone-Wales and flower defects shown in (a) and (b) are purely structural defects with an atomic density identical to the pristine lattice. In contrast, double vacancy (c) and defects containing additional atoms (d) show density deficiency and excess, respectively. The network on top corresponds to the atomic positions in graphene and/or the positions silicon atoms in HBS. Note that the AC-HRTEM images in (c), in addition to the reconstructed divacancy schematically shown on the top row, show two merged defects of the same type, both for graphene and HBS.

While analysing deviations from perfect crystalline order, it is instructive to consider first point defects, specifically, topological defects, such as the SW defect^10^ in graphene, which can be constructed by rearranging atomic bonds without removing/adding atoms. In the SW transformation, two neighbouring nodes in the hexagonal network are rotated by 90° around their midpoint. In graphene the nodes are two neighbouring C atoms (see e.g. Refs. 18, 19 for AC-HRTEM images of the defect), in HBS four Si atoms (two in each layer) with the neighbouring O atoms. The application of this transformation to HBS is schematically presented in Fig. 2, and we present an AC-HRTEM image of the defect in Fig. 3 with other defects and a comparison to graphene images of similar structures. In Fig. 3b, we show the so-called *flower* defect^20^,^21^, which can be formed via six successive bond rotations, and can be considered a small grain with 30° rotation with respect to the lattice surrounding it. SW-type defects can appear during the material growth or induced by impacts of energetic electrons, as discussed below.

Other defects are formed when structural units are removed from the lattice. Since single vacancies are very rare in AC-HRTEM observations of graphene^19^,^22^, most likely due to their high reactivity and low stability against electron beam damage, we concentrate our analysis on double vacancies, with all bonds saturated. One example of a double vacancy is presented in Fig. 3c. This 5555-6-7777 defect (occasionally referred to as the *butterfly* defect^23^) can be created by removing two neighbouring structural units and then applying two bond rotations on the structure. Schematic illustrations of other examples are given in the Supplemental Material. These double vacancy structures have been extensively studied in graphene by theory and experiment^9^,^24^ with techniques similar to those employed here. Also added structural units lead to the formation of non-hexagonal rings in a lattice composed otherwise of hexagons. For example, adding two units gives rise to the *inverse* SW defect^25^, which can be further transformed via rotations, producing for example the defect shown in Fig. 3d.

To estimate the relevance and driving force behind the formation of the defects, we calculate the formation energies *E_f_* of selected defect configurations (see Table I). We define *E_f_* as 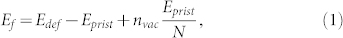
where *E_def_* is the energy of the lattice with the defect, *E_prist_* is the energy of the corresponding pristine structure, *n_vac_* is the number of missing units (atoms) and *N* is the total number of units in the system (thereby, *E_prist_*/*N* is the chemical potential of the structural units in the pristine system).

Our DFT calculations were carried out for structures with up to 2000 atoms, whereas much larger system sizes were used with CFF. By using the more accurate, but also more computationally expensive, DFT approach as a benchmark for the CFF data, we noticed that the CFF method accurately reproduces all the trends studied, albeit the formation energy is systematically higher than the DFT values, as seen in Table I. Therefore, CFF allows us to scale up the system sizes beyond what is accessible with the DFT approach without compromising the accuracy of our description.

The most striking difference between the *E_f_* for defects in graphene and in HBS, as shown in Table I, are the lower values in HBS for any defects involving rotations. For example, the 5-8-5 divacancy, which can be created by simply removing two structural units and saturating the bonds, has a formation energy of about 9 eV in HBS, but only ~ 7.5 eV in graphene. However, already after one rotation (555-777), the situation is reversed. After a second rotation (5555-6-7777), the trend has become even clearer. Similarly, although already SW has a lower *E_f_* in HBS than in graphene, adding six more rotations *decreases E_f_* in HBS (2 → 1 eV), where as it *increases* in graphene (~ 5 → 7 eV). According to Ref. 26, higher defect concentrations further decrease the *E_f_* in HBS when supercell relaxation is allowed (in our case the supercell remained fixed because we were interested in isolated defects).

**Figure 4:**
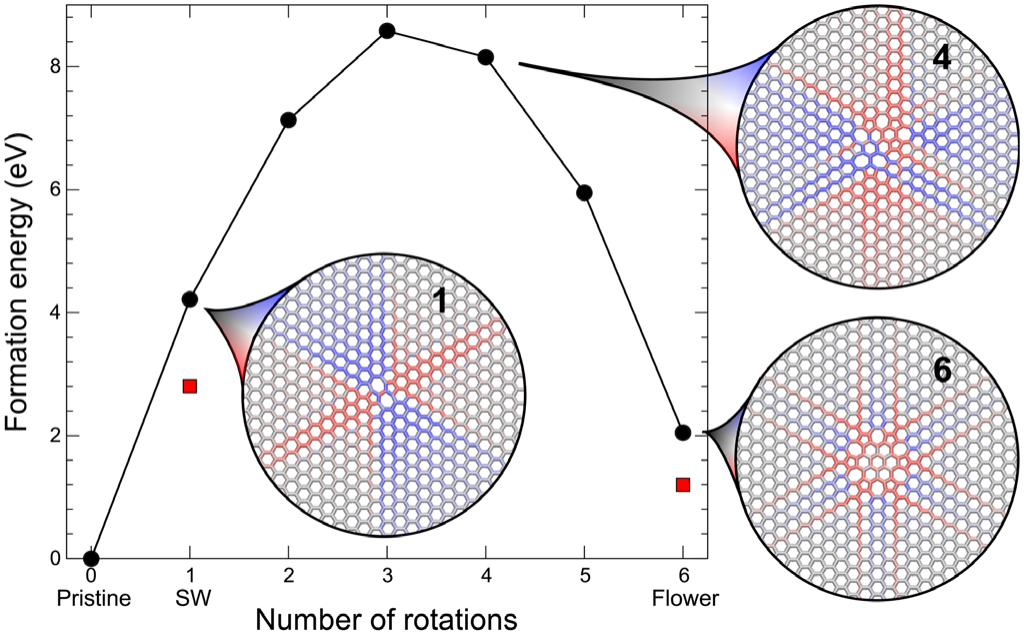
Total energy as a function of successive Stone-Wales transformations in HBS for crystalline structure to the flower defect. Black circles denote CFF and red squares DFT calculations. The insets shows strain fields around the SW defect (1 rotation), an intermediate structure after 4 rotations and the flower defect (6 rotations). Blue colour denotes expansion and red contraction in the distances between the neighbouring structural units (only connecting lines between the units are displayed).

To understand the reason for this behavior, we calculate *E_f_* for all steps between the crystalline HBS structure and the flower defect (Fig. 4). The behaviour is very similar to that previously seen in graphene^27^: at first, *E_f_* increases with every rotation, then decreases from the fourth rotation, until the flower defect is reached. As can be seen from Fig. 4, CFF predicts consistently higher values than DFT, but the relative differences between different configurations are similar for the two methods. In the insets of Fig. 4, we use the deviation from the pristine lattice of the Si-Si distances to visualise the strain fields around three different configurations between the crystalline structure and the flower defect. Red colour indicates positive strain (contraction), blue negative strain (expansion) and grey corresponds to zero strain. Strain fields for all configurations in Fig. 4 and a more extensive technical discussion are given in the Supplemental Material. The strain field in the last configuration has a much shorter spatial extent than the intermediate defects, illustrating that the flower defect is less constrained by embedding in the pristine lattice, which explains the low *E_f_* for this structure. The long ranged strain fields in HBS around the SW defect were also studied in Ref. 8.

### The SW transformation in HBS

**Figure 5:**
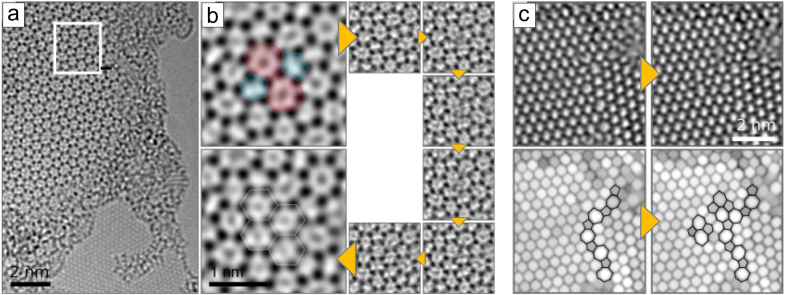
80 kV AC-HRTEM images of Stone-Wales transformations in HBS. (a) Overview image of a disordered area of HBS. (b) Higher magnification image of the area marked in panel (a) along with a series of subsequent images of the same area showing intermediate atomic configurations during annihilation of a SW. (c) Two examples of SW transformations in crystalline HBS. The upper row: original AC-HRTEM micrographs. Lower row: same images with maximum filtering^28^ applied for better visibility of the defect structures.

In the case of graphene, the mechanism of bond rotation is relatively simple since only two atoms are involved. However, the associated energy barrier is so high that in practice their formation through thermal activation is impossible^11^. However, the rotation can be driven by an electron impact during imaging in a AC-HRTEM device^18^. Due to the much more complicated structure of HBS, one could a priori expect that the corresponding transformation would be entirely impossible. Nevertheless, careful study of subsequent AC-HRTEM images clearly show that they occur. As an example, a reverse SW transformation in vitreous bilayer silica is shown in a series of images in Fig. 5, along with two examples of the appearance of SW defects in an initially crystalline area. Such a process has been recently studied in Ref. 8, where the projected positions in the *xy* plane of the Si atoms in some intermediate positions were tracked. AC-HRTEM allows for direct observation of atomic rearrangements, but details of the transformation are still unclear, primarily due to the experimental time resolution (ca. 1 second), the lack of information of the out-of-plane motion of the Si atoms and the poor contrast of the oxygen atoms. Beyond doubt, however, is that the SW transformation in HBS is a complex process involving several steps where the atoms gradually shift around from the 5-7 to a 6-6 geometry and not the simple trajectory schematically outlined in Fig. 2 with a rigid rotation of a dimer-like structure.

In order to get insight into the transformation process stimulated by the beam, we calculated the displacement threshold *T_d_* (minimum kinetic energy assigned to an atom required for it to sputter away from the system) of O and Si atoms in HBS using DFT-based molecular dynamics, similar to earlier calculations for graphene^29^, BN^30^ and transition metal dichalcogenides^31^. We obtained *T_d_* = 16.8 eV for Si and *T_d_* = 11.6 eV for O. These values indicate (see the Supplementary Information) that only O atoms will be displaced during imaging by the 80 keV electron beam used in our experiments, which confirms the assertion of Ref. 8 that oxygen deficiencies are an important factor driving topological transformations of the lattice. We further calculated *E_f_* for a SW defect in the presence of an oxygen double vacancy, removing the two oxygen atoms that form the bridge of the central dimer in Fig. 2, and this indeed decreases *E_f_* by about 0.5 eV. This indicates that transition states involving oxygen deficiencies can have lower energy barrier for the SW transformation, leading to an increased number of bond rotations. The vacancies created can be filled again during the experiment by atoms coming from outside the area of interest.

### Grain boundaries

**Figure 6:**
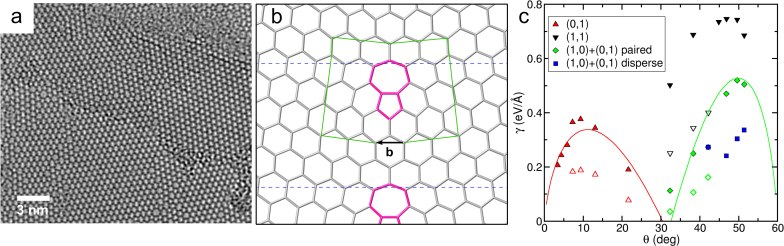
(a) 80 kV AC-HRTEM image of grain boundaries in crystalline HBS. (b) Example of a (1,0) grain boundary in HBS. The dashed blue lines indicates the boundary of the periodically repeating cell. The Burgers' vector, (b) for the (1,0) dislocation is indicated in the figure along with a corresponding Burgers' circuit in green. Only connecting lines between the structural units are displayed with non-hexagonal rings highlighted in pink. (c) Formation energy of different grain boundaries in HBS. CFF calculations are shown as filled symbols and DFT calculations as open symbols.

As evident from the AC-HRTEM images, Figs. 5(c) and 6(a), crystalline HBS frequently consists of grains separated by boundaries reminiscent of those in graphene. The majority of the grains are oriented with respect to each other at an angle of around 30°, and overall, the average sizes of the grains are much smaller than the typical grain sizes in graphene grown by chemical vapour deposition. To understand the reason for abundance of grain boundaries (GBs) and their preferential orientation, we considered various extended topological defects in HBS. As for graphene^32^, these one-dimensional defects can be used as idealized models of grain boundaries, which ignore the curved shapes of the GBs normally seen in experiments. The geometry of one such GB studied is shown in Fig. 6(b), where a dislocation core in the form of a 5-7 point defect adds one extra column of hexagons in the cell. Using notations introduced in Ref. 32, this is a (1,0) dislocation. When the cell is repeated along the *y* axis, the effect is to create a boundary between two sheets of HBS at an angle to the center line of the dislocation which depends on how closely spaced the dislocation cores are. To get larger angle grain boundaries, we can also construct so-called (1,1) and (0,1) + (1,0) boundaries, both of which insert two extra rows of hexagons per dislocation, and these are characterised by an angle *θ* = 60° − *θ*′.

The formation energy of grain boundaries, γ, as function of the angle *θ* is shown in Fig. 6c, as calculated by the CFF and DFT methods. The CFF values are found to be consistently about twice as high as those calculated with DFT, so that scaling the CFF results by a factor of 0.5 gives a near-perfect agreement with DFT. In comparison with the results of Yazyev and Louie for graphene^32^, the calculated formation energy curves are markedly less smooth, more reminiscent of the results for MoS_2_ by Zou et al.^33^. We expect that this is due to the internal structure of the hexagonal network links, which allow for additional relaxation due to the possibility to rotate the tetrahedra in some geometries. This applies in particular to situations which correspond to bond contractions in graphene, as was shown in Ref. 4 to explain the anomalously soft behaviour of HBS on contraction. It should also be noted in this context that we only considered grain boundary structures analogous to those found in graphene, and do not account for the possibility of O deficient grain boundaries with direct Si-Si bonds, similar to direct Mo-Mo and S-S bonding recently seen to be important for MoS_2_^33^,^34^,^35^.

For the (1,0) and (1,0) + (0,1) grain boundaries, we also perform a fit of the calculated values to a Read-Shockley curve^36^ for the grain boundary energy, as derived from continuum theory. The equation is of the form 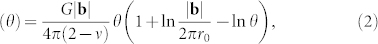
where **b** is the Burgers' vector, *G* the shear modulus, *ν* Poisson ratio, and *r*_0_ is related to the energy of the dislocation core. Since it is difficult to unambiguously decide how the 3D shear modulus should be calculated for a 2D system (the dimensionless Poisson ratio is not sensitive to the precise definition of the height of our system, and we can use our calculated 2D value for this quantity), we produce the two solid curves shown in Fig. 6c by fitting one *G* and two independent *r*_0_ parameters simultaneously for the two curves. The resulting values for *r*_0_ are 4.33 Å and 8.31 Å for the (1,0) and (1,0) + (0,1) dislocations, respectively. The fitted value for the shear modulus, 440 GPa, is close to the value obtained from direct calculations by dividing the calculated 2D value by the height of the bilayer, to get a volume normalisation suitable for comparison with the Read-Shockley theory, showing that the analysis is meaningful (see the Supplementary Information for further details). The ratio of the Burgers' vector to *r*_0_ controls when the initial linear increase of γ(*θ*) will be overtaken by the logarithmic decrease of the last term in Eq. 2. We note that **b**/*r*_0_ ≈ 1 for the (1,0) dislocations in HBS compared to **b**/*r*_0_ ≈ 2 calculated for graphene^32^, indicating the much lower GB formation energies in HBS at larger angles. All the investigated grain boundary types go towards low formation energies near 30°, in line with the experimental observations.

### Effects of strain and Haeckelite structures

**Figure 7:**
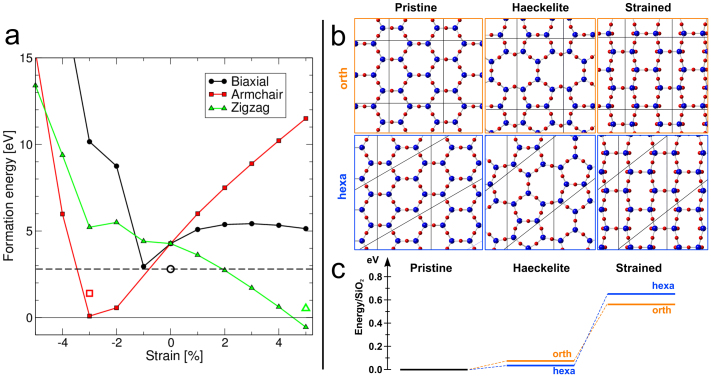
Effect of strain on the formation energy of SW. (a) The dependence of the SW defect formation energy on various types of strain. Filled and open symbols denote CFF and DFT calculations, respectively. (b) Two Haeckelite structures produced from crystalline HBS (leftmost panels) by single SW transformations in an orthorhombic cell (upper panels, orange) and an initially hexagonal cell (lower panels, blue), four times larger than the primitive cell. Rightmost panels depict the crystalline structure subjected to a strain similar to that resulting from the Haeckelite transformation. (c) Diagram showing the energy differences for the structures in panel b, illustrating the energy gain in formation of the Haeckelite structure at large strains.

During TEM imaging, our HBS samples shrink constantly, which indicates high strain in the structure. Therefore, we extended the study of *E_f_* to strained structures. Our simulations showed that the external strain strongly affects the formation energy of the defect, as shown in Fig. 7(a), where *E_f_* of the SW defect is plotted as a function of strain. The effect of stretching the lattice along the zigzag direction is reminiscent of a similar effect seen in carbon nanotubes^37^, but in HBS, *E_f_* also decreases on contraction along the armchair direction. The reason for this can be understood from strain field configuration near the SW (Fig. 4, inset): the defect expands the lattice along the zigzag direction (i.e., the bonds are contracted) and contracts it along the armchair direction (the bonds are stretched), which means that in presence of strain, local or global, the defect formation energy will be drastically altered. Therefore, external strain reduces *E_f_* of the SW and can even make it negative, so that strain and local oxygen deficiency may both contribute to the observed transformations.

As an extreme limit, both in terms of high defect concentration and strain, we also studied the formation energy of the so-called Haeckelite structures, as suggested earlier for graphene^38^,^39^. These are 2D structures reminiscent of graphene or HBS but consisting of 5- and 7-rings instead of hexagons. We obtained two different Haeckelite structures by performing a SW transformation in two unit cells 4 times larger than the primitive hexagonal cell, the smallest possible supercells to be able to accommodate an SW defect, as shown in Fig. 7(b). The resulting energies for the two structures are 73 meV/SiO_2_ (orthorhombic) and 33 meV/SiO_2_ (monoclinic) above the crystalline hexagonal lattice (see Fig. 7(c)). These energies are very similar to those reported by Lichtenstein et al. (57–99 meV/SiO_2_)^26^ for four small systems modeling the vitreous state of HBS. The cost of making a Haeckelite structure is much smaller in HBS than in graphene, which may explain why 2D amorphous silica can be grown on metals, while only special irradiation treatment gives rise to coherent amorphisation of graphene^9^.

## Conclusions

Having analysed various defects in graphene and HBS, two systems consisting of hexagonal networks, we have shown that the behaviour of defects in these two materials is qualitatively very similar, despite the more complicated structure of HBS, involving different types of atoms. This holds true for point defects, like the SW defect, or extended defects, like grain boundaries. This is in contrast to the more complex situation of defect structures in 2D systems like boron nitride^40^ and transition metal dichalcogenides^33^,^35^, which are both multi-component systems of trigonal, not hexagonal, symmetry. In such trigonal systems, the network links are more strongly constrained, so that the Stone-Wales transformation is much less likely due to the formation of homonuclear pairs. For instance, SW defects have never been observed in 2D boron nitride, in spite of experimental efforts^41^,^42^. In conjunction with the fact that both *sp*^2^-hybridised carbon and bilayer silica can form amorphous structures and possibly Haeckelites, our results strongly suggest that the defect structures will be very similar in any hexagonally linked 2D system, possibly also in epitaxial transition-metal networks^13^, irrespective of the complexity of the structural units of the hexagonal network.

We note that all the investigated grain boundary types go towards low formation energies near 30° angle, which is in line with the small value of the formation energy of the flower defect, and the experimental observations. In these defects, the contribution to the formation energy due to long-range elastic fields is minimized similar to the case of graphene. This is due to the fact that constituent dislocations (5-7 pairs) are closely packed, leading to an efficient cancellation of elastic fields. However, in contrast to graphene, HBS allows for additional relaxation in the dislocation cores due to the possibility of rotating tetrahedral building blocks, which is reflected in a significantly smaller ratio of the Burgers' vector to the dislocation core^32^. This ultimately explains the tendency of HBS towards hosting ~30° grain boundaries and flower defects as well as low formation energies for Haeckelites and realization of the vitreous phase.

The most important difference between defects in silica and graphene concerns the formation and dynamics of defects, which is significantly more complex in HBS due to the much larger number of atoms involved. We find that the momentum of an electron is never transferred to one complete structural unit of the silica lattice but to single atoms within this unit. Most surprisingly, the SW transformation, is still possible and observable in silica under electron irradiation, similar to graphene. Our calculations indicate that these processes can be driven by electron irradiation through the lowering of the barriers for displacement of atoms due to vacancies from sputtered oxygen atoms and the strain that these vacancies induce.

## Methods

The HBS films were produced on top of a graphene substrate using the low pressure CVD process described in Ref. 3. In short, the graphene + HBS films were grown on Cu foils attached to a quartz substrate placed in a quartz tube, using hexane as the precursor gas. As explained in Ref. 3, the growth process aimed at producing graphene, and the formation of the 2D silica was an accidental side product. As previously shown in Ref. 3, the HBS is very weakly bonded to the graphene, and can in practice be seen as free-standing. The grown films were transferred onto commercial TEM grids (Quantifoil) by etching away the Cu foil in 15% nitric acid and fishing the floating film onto the grid. After the transfer, the samples were rinsed in distilled water and dried in dry nitrogen. The samples were heated to 200°C for 10 minutes before the TEM investigations.

The HRTEM imaging was performed in a FEI Titan 80–300 microscope equipped with an image-side spherical aberration corrector. The microscope was operated at 80 kV, and the extraction voltage of the field emission gun was lowered to 2 kV in order to reduce the energy spread of the electron source. The spherical aberration was set to approximately 20 *μ*m.

DFT calculations were performed using the projector augmented-wave method^43^ as implemented in the VASP package^44^,^45^,^46^. Brillouin zone sampling was done on a reciprocal space mesh with a spacing of 0.2Å for the smaller Haeckelite cells and for the large supercells used for calculation of defect formation energies, just the Γ point were sufficient. In the direction perpendicular to the HBS plane was padded with vacuum to give an interplanar distance of 20 Å to describe an isolated single layer. The classical force field (CFF) calculations were carried out using with the molecular dynamics package PARCAS^47^, with a potential by Watanabe et al.^48^ to describe the Si-O system. For the CFF calculations, the calculation cell had periodic boundary conditions only in the 2D plane.

The defect formation energies were always calculated as the energy difference between a cell with a defect and a cell without a defect of the same size, to ensure that exactly the same plane basis set was used in both cases. By this procedure, the formation energies were converged to within 0.01 eV with a plane wave cutoff of 400 eV. We consider free-standing 2D SiO_2_ without accounting for the substrate (metal or graphene), as the vdW interaction between the substrate and bilayer is very weak and should not have any effects on defect energetics governed by covalent bonding. Like any other form of silica, HBS in all forms studied in this paper is a wide gap insulator and the only change of the electronic properties worthy of note is a small reduction in the band gap when introducing defects in the system. Further technical details on the convergence of supercells are presented in Section I of the Supplementary Material.

## Author Contributions

T.B. performed calculations for all defect structures with assistance from O.L. in the CFF calculations T.B. prepared and finalised figures. J.K. carried out the displacement threshold calculations. S.K. performed the TEM experiments. S.K. and O.L. analysed the HRTEM images. O.V.Y. supplied the grain boundary models and analysed the grain boundary results. Samples were grown by A.S., V.S. and J.S. at MPI Stuttgart. T.B. and A.V.K. wrote the main manuscript. All authors commented on the manuscript. U.K. and A.V.K. proposed and supervised the project.

## Supplementary Material

Supplementary Information

## Figures and Tables

**Table 1:** Defect formation energies in HBS compared to the corresponding structures in graphene. The values for graphene are from Ref. 11, with the exception of the flower defect from Ref. 20. Schematic illustrations of the defect structures are given in the Supplemental Material

	Defect formation energy (eV)		
Defect	Graphene	HBS (DFT)	HBS (CFF)
Stone-Wales (55-77)	4.5–5.3	2.8	4.2
Flower	7.0	1.2	2.0
5-8-5	7.2–7.9	9.0	10.1
555-777	6.4–7.5	5.7	6.5
5555-6-7777	7	4.8	5.1
