# Supplementary material for: Defects in bilayer silica and graphene: common trends in diverse hexagonal two-dimensional systems
Source: Sci Rep. 2013 Dec 16;3:3482. doi: 10.1038/srep03482 (PMC3863822; doi:10.1038/srep03482)
Supplement: Supplementary Information [file srep03482-s1.pdf]

Supplemental Material for "Defects in bilayer silica and graphene: common trends in diverse hexagonal two-dimensional systems"

Torbjörn Björkman,[1] Simon Kurasch,[2] Ossi Lehtinen,[2] Jani Kotakoski,[3,4] Oleg V. Yazyev,[5] Anchal Srivastava,[6,7] Viera Skakalova,[6,4] Jurgen H. Smet,[6] Ute Kaiser,[2] and Arkady V. Krasheninnikov[1,3]

[1] *COMP/Department of Applied Physics, Aalto University School of Science, P.O. Box 11100, 00076 Aalto, Finland*
[2] *Electron Microscopy Group of Materials Science, University of Ulm, Germany 89081*
[3] *Department of Physics, University of Helsinki, P.O. Box 43, 00014 Helsinki, Finland*
[4] *Faculty of Physics, University of Vienna, Boltzmanngasse 5, 1190 Wien, Austria*
[5] *Institute of Theoretical Physics, École Polytechnique Fédérale de Lausanne (EPFL), CH-1015 Lausanne, Switzerland*
[6] *Max Planck Institute for Solid State Research, Stuttgart, Germany 70569 and*
[7] *Department of Physics, Banaras Hindu University, Varanasi, India 221005*

(Dated: October 22, 2013)

DETAILS OF DEFECT CALCULATIONS

Divacancies

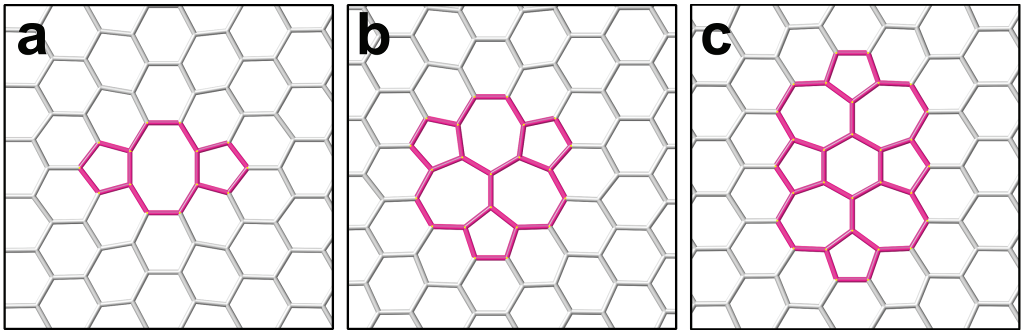

FIG. 1. Three double vacancy type defects, which have the same morphologies in HBS and graphene. (a) 5-8-5, (b) 555-777 and (c) 5555-6-7777. The lines correspond to C-C bonds in graphene and connect neighboring silicon atoms in the case of HBS. Non-hexagonal rings are highlighted in pink.

Three different classical force fields (CFF) for SiO_2 were tested for suitability against DFT results. These were the force fields of Watanabe et al.[1], Ohta et al.[2], using the PARCAS code[3] and a Tersoff potential[4, 5], using the LAMMPS package[6]. The results for defect formation energies are given in Table I. The Tersoff potential is clearly unsuitable in this case, as it gives negative formation energies of two of the defects and does not follow the DFT trend well. The Watanabe and Ohta potentials produce similar trends, which both follow the trends of the DFT calculations well, but the better overall agreement make us prefer the Watanabe potential in the CFF calculations.

TABLE I. Defect formation energies (in eV) of the SW defect and selected divacancies in HBS from different classical force field models compared with DFT.

Defect	DFT	Watanabe	Ohta	Tersoff
Stone-Wales (55-77)	2.8	4.2	8.0	2.9
5-8-5	9.0	10.1	18.1	1.3
555-777	5.7	6.5	11.0	-1.1
5555-6-7777	4.8	5.1	8.5	-1.4

Supercell convergence

Figure 2 shows the convergence of a Stone-Wales defect in hexagonal bilayer silica (HBS) as function of the distance, d, between the periodic images, along with fits to the function $E(d) = E_F + a \cdot e^{-b \cdot d}$, with a, b and E_F being constants fitted to the datapoints, calculated using density functional theory (DFT, in red) and a classical force field (CFF, in black). E_F is the asymptotic value which gives the formation energy of a single defect. The black curve for the CFF calculations were fitted to only the first 5 datapoints, which was seen to give almost no difference compared with doing fitting to all points, thus validating the extrapolation procedure. The strain field interaction is remarkably long ranged — not until the spacing exceeds 5 nm we start to see a reasonably isolated defect.

ELASTIC PROPERTIES

The strain vs. energy per unit area, shown in Figure 3 has a somewhat peculiar form, and is drastically different from the case of graphene, by being softer on the compression side than on the expansion side, due to the possibility for the oxygen tetrahedra to rotate, as has been discussed elsewhere[7]. Near zero strain, however, the lattice behaves

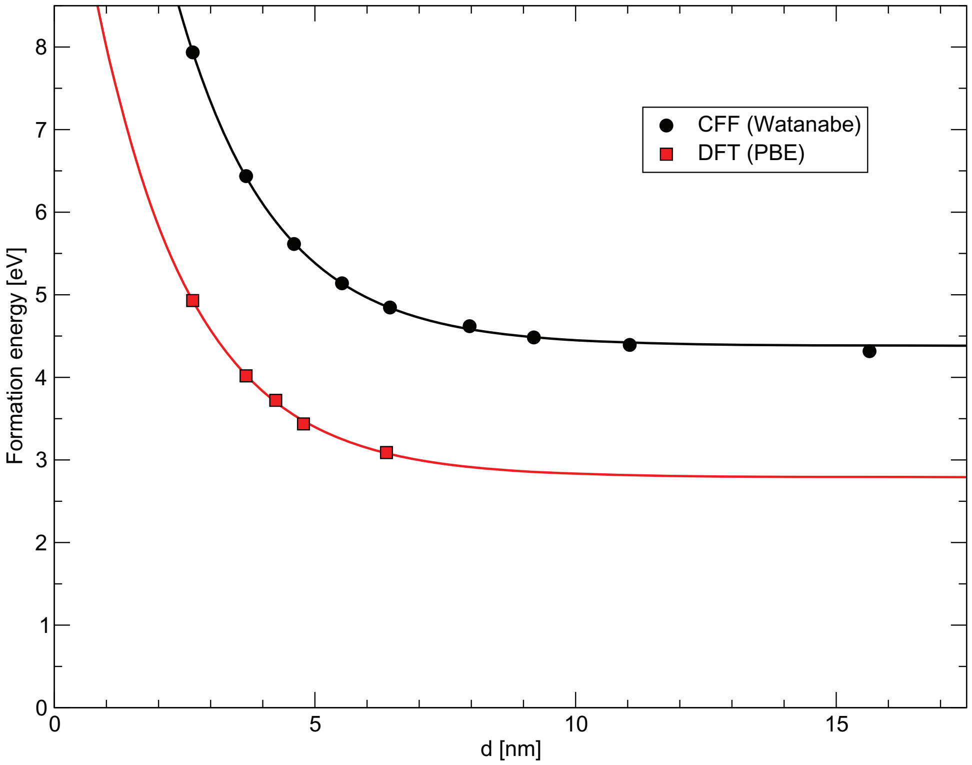

FIG. 2. Convergence of the formation energy of a Stone-Wales defect as function of the supercell size. The d axis gives the distance between the periodic images in nm. The black and red symbols are datapoints calculated with CFF and DFT, respectively, and the continuous lines are fitted to these values as described in the text.

harmonically and in this region we obtain for HBS the values for the 2D Youngs's modulus and Poisson ratio, $E = 260$ N/m and $\nu = 0.328$, respectively, both fairly close to values reported for graphene[8].

The shear modulus is given by the Young's modulus and the Poisson ratio as

$$G = \frac{E}{2(1+\nu)}, \tag{1}$$

which yields $G = 98$ N/m for HBS. To get a normalization per volume, suitable for comparison to the value of 440 GPa obtained by fitting of the grain boundary formation energies from CFF calculations to the Read-Shockley expression, we simply divide this by the height of the HBS (4.60 Å). This yields $G = 213$ GPa, almost precisely a factor 2 from the result obtained by fitting to the Read-Shockley expression. Since the Read-Shockley expression is linear in the shear modulus, this is the same factor of 2 difference between the DFT and CFF results for the grain boundary formation energies. This demonstrates once again that while the CFF energies do not quantitatively match the DFT results, the results are qualitatively consistent.

STRAIN FIELD VISUALIZATION

The strain field in a material is normally defined as the deviation of the lattice points of the strained system from those of the unstrained system. One way to study this is to visualise the deviation of the Si-Si distances from those of the pristine HBS lattice, as done in Figure 4 for the set of defects shown in Figure 5 of the main paper. Since our primary concern here is to demonstrate the long range behaviour of the strain fields, this form is somewhat more convenient, since it does not overemphasise the very large deviations of the positions of the atoms that constitute the defect itself and it would be equally applicable for defects which do not have the same number of structural units as the pristine lattice.

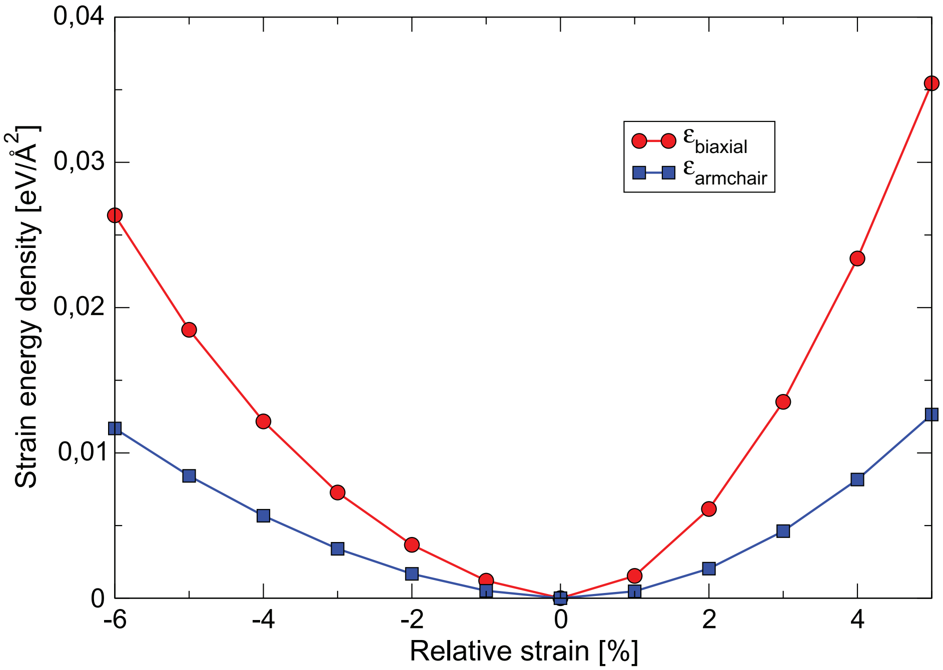

FIG. 3. The strain-energy curves for biaxial in-plane strain and strain along the armchair direction of the lattice calculated with DFT. Straining the lattice along the zigzag direction produces a curve virtually identical to the armchair curve for the rather small strains considered here. The strain curve has the peculiar property that, for the small strains shown here, it is softer on the compression side, due to the possibility for the oxygen tetrahedra to rotate with respect to each other, which is less costly energetically than stretching of the bonds.

[1] T. Watanabe, H. Fujiwara, H. Noguchi, T. Hoshino, and I. Ohdomari, Jpn. J. Appl. Phys. **38**, L366 (1999).
[2] H. Ohta and S. Hamaguchi, J. Chem. Phys. **115**, 6679 (2001).
[3] K. Nordlund, Comput. Mater. Sci. **3** (1995).
[4] S. Munetoh, T. Motooka, K. Moriguchi, and A. Shintani, Comput. Mater. Sci. **39**, 334 (2007).
[5] P. Y. Huang, S. Kurasch, J. S. Alden, A. Shekhawat, A. A. Alemi, P. L. McEuen, J. P. Sethna, U. Kaiser, and D. A. Muller, Science **342**, 224 (2013).
[6] S. Plimpton, J. Comput. Phys. **117**, 1 (1995).
[7] F. Ben Romdhane, T. Björkman, J. A. Rodríguez-Manzo, O. Cretu, A. V. Krasheninnikov, and F. Banhart, ACS Nano **7**, 5175 (2013).
[8] E. Cadelano, P. L. Palla, S. Giordano, and L. Colombo, Phys. Rev. Lett. **102**, 235502 (2009).

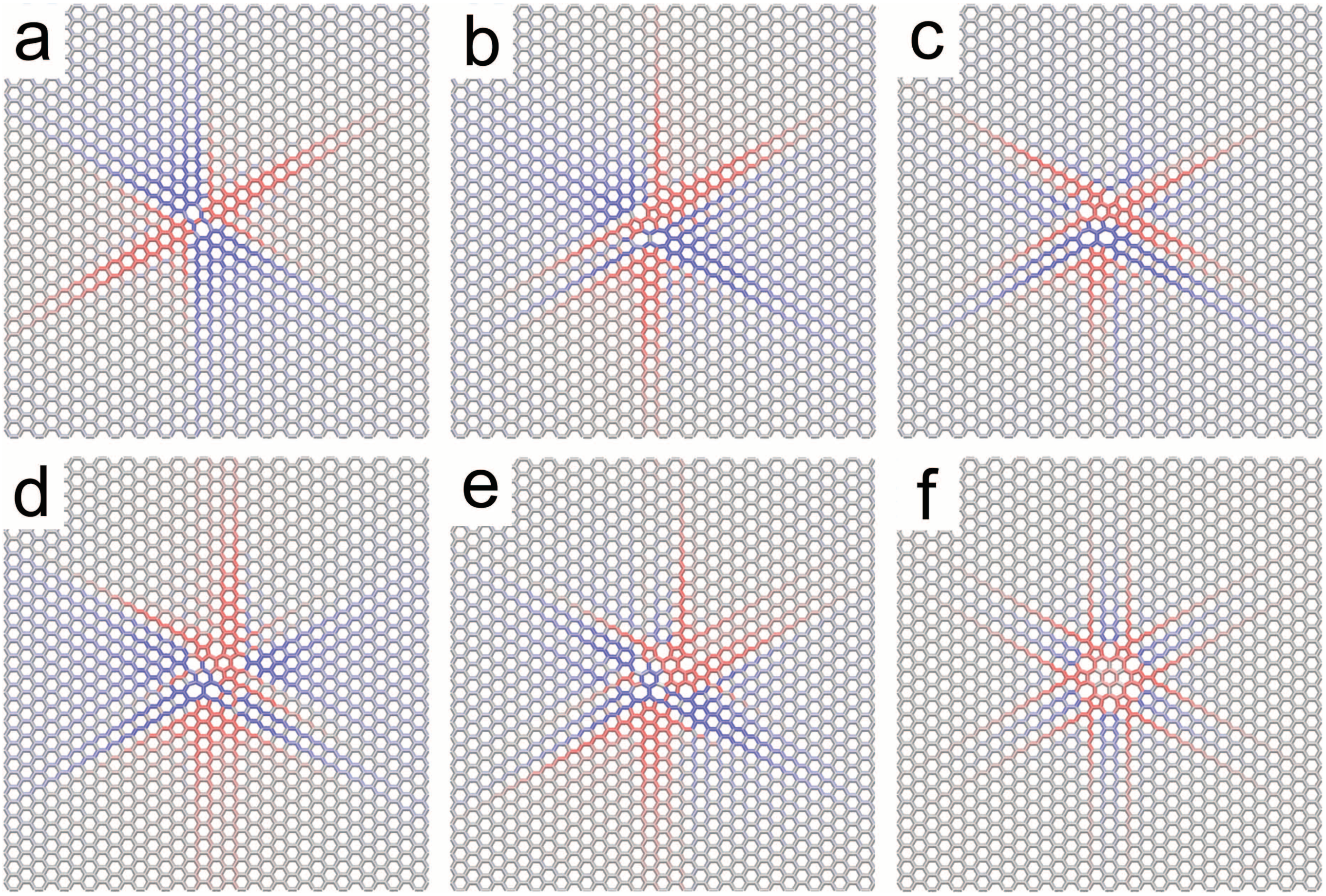

FIG. 4. Visualization of strain fields by means of the deviations of Si-Si distances for the series of isolated defects from SW defect to flower defect shown in Figure 5 of the main paper. Panel (a) shows the Stone-Wales defect, Panel (f) the flower defect and Panels (b)-(e) the successive steps in between.

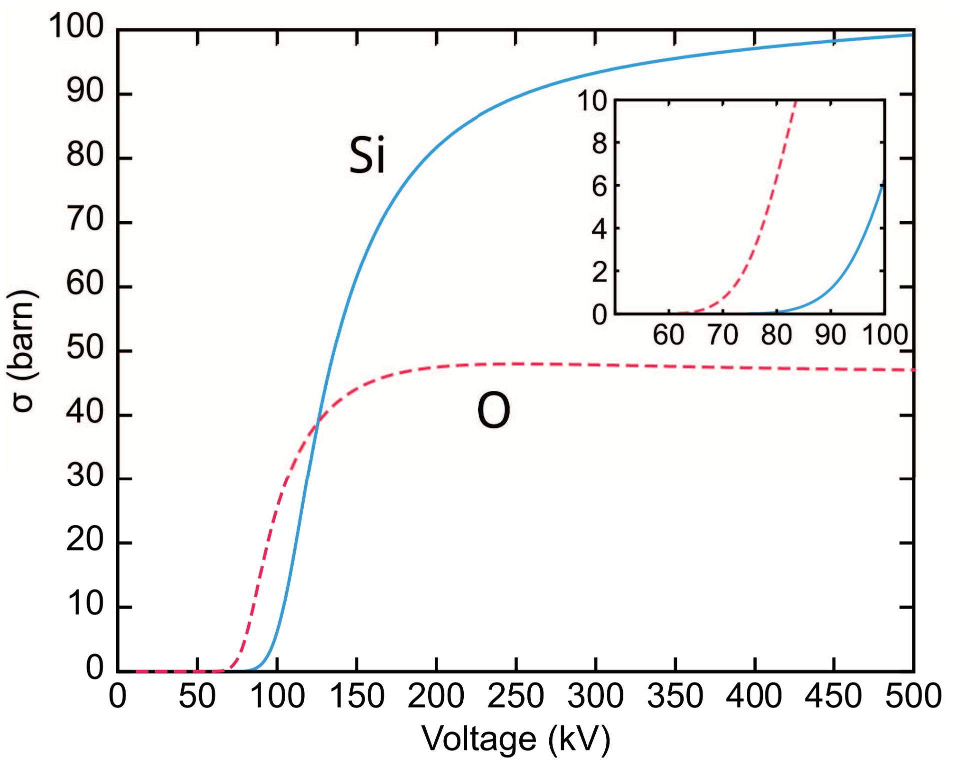

FIG. 5. Calculated cross-section for atom displacement as a function of electron energy. The cross section drops to zero at about 90 kV (silicon) and 70 kV (oxygen), indicating that only O atoms can be displaced by a 80 keV electron beam.
